# Valoración de necesidades formativas de nivel básico en cuidados paliativos en enfermeras de atención primaria en España

**DOI:** 10.1016/j.aprim.2022.102344

**Published:** 2022-04-27

**Authors:** Isidro García-Salvador, Encarna Chisbert-Alapont, Amparo Antonaya Campos, Jorge Casaña Mohedo, Clara Hurtado Navarro, Silvia Fernández Peris, José Bonías López, María Luisa de la Rica Escuín

**Affiliations:** a Enfermería, Servicio de Oncología, Departamento de Salud Dr. Peset, Valencia, España; b Fundación para el Fomento de la Investigación Sanitaria y Biomédica de la Comunitat Valenciana (Fisabio), Valencia, España; c Grupo de Investigación INCUE, Departamento de Salud Dr. Peset, Valencia, España; d Enfermería, Servicio de Hematología, Departamento de Salud La Fe, Valencia, España; e Dirección de Atención Primaria, Departamento de Salud Dr. Peset, Valencia, España; f Departamento de Enfermería, Facultad de Medicina y Ciencias de la Salud, Universidad Católica San Vicente Mártir, Valencia, España; g Adjunta de Docencia, Departamento de Salud Dr. Peset, Valencia, España; h Asociación de Psicooncología Carena, Departamento de Salud Dr. Peset, Valencia, España; i Enfermería, Centro de Salud de San Marcelino, Departamento de Salud Dr. Peset, Valencia, España; j Enfermería, Grupo de Investigación del Cuidado en el proceso de final de vida, Instituto para la Investigación en Salud de Aragón, Zaragoza, España

**Keywords:** Enfermería, Atención primaria, Cuidados paliativos, Formación, Nursing, Primary care, Palliative care, Training

## Abstract

**Objetivo:**

Valorar la formación, la percepción de preparación y las necesidades formativas en cuidados paliativos (CP) teóricas y prácticas de las enfermeras de atención primaria en España.

**Diseño:**

Estudio descriptivo transversal.

**Emplazamiento:**

Centros de atención primaria en España con acceso online.

**Participantes:**

Enfermeras de atención primaria en España durante enero y febrero de 2021. De las 344 respuestas, 339 cumplían criterios de inclusión.

**Mediciones principales:**

Se analizaron variables sociodemográficas, formación en CP, necesidades de formación mediante cuestionario online de Google Forms e Instrumento INCUE. Se realizaron análisis descriptivos y se compararon los resultados mediante test de simetría exacto y test de Mann-Whitney.

**Resultados:**

Mayoritariamente mujeres (82,6%) con una media de edad de 45,5 años. El 86,1% de las enfermeras encuestadas tenían formación en CP, siendo básica en el 45,4%. Solo el 40,5% se sienten bastante o muy preparadas para cuidar de pacientes paliativos. Demandaban mayor formación en psicoemocional y duelo y afrontamiento de pérdidas. Superaron el bloque teórico el 83,76%, frente el 43,36% del práctico, detectándose mayores necesidades formativas en este último (p < 0,001). Las proporciones de capacitados variaban en función del nivel formativo.

**Conclusiones:**

La formación enfermera en CP en atención primaria continúa siendo deficitaria sobre todo en su aplicación práctica, siendo necesaria formación dirigida para que repercuta en el cuidado de las personas con necesidades paliativas y sus familias.

## Abstract (en)

**Aim:**

Asses training, perception of readiness and training needs in palliative care (PC) theoretical and practical of primary care nurses in Spain, through descriptive cross-sectional study.

**Setting:**

Primary care nurses in Spain with online access.

**Participants:**

Primary care nurses in Spain, January and February 2021. 344 responses, 339 met the inclusion criteria.

**Main neasurements:**

Sociodemographic variables, PC training, training needs were analyzed. Through Google Forms online questionnaire and INCUE Instrument. Descriptive analyses were performed and the results were compared using the exact symmetry test and the Mann-Whitney test.

**Results:**

82,6% women, with an average age of 45.5 years. 86.1% of the nurses had training in PC, been basic in the 45.4%. Only 40.5% feel quite or very prepared to take care for palliative patients. Nurses demanded more training in psycho-emotional and grief and coping with losses. 83.76% passed the theoretical block compared to 43.36% of the practical, detecting higher training needs in the last (*P* < .001). The passed rates varied depending on the educational level.

**Conclusions:**

Nursing training in PC in primary care continues to be deficient, especially in practical application. Targeted training is necessary to have an impact on the care of people with palliative needs and their families.

**Lo conocido sobre el tema**

- La importancia de la atención primaria en el cuidado de las personas con necesidades paliativas y de sus familias y el papel destacado imprescindible de las enfermeras.
- Las diferencias formativas en cuidados paliativos de las enfermeras de atención primaria en España.

**Qué aporta este estudio**

- Los conocimientos teóricos mayoritariamente no implican la aplicación práctica de los mismos. Esto supone que, a pesar de poseer los conocimientos necesarios para proporcionar cuidados paliativos, estos no son recibidos por las personas a las que van dirigidos.
- La necesidad de formación específica dirigida a las enfermeras de atención primaria para mejorar la atención de las personas con necesidades paliativas y sus familias.

## Introducción

El domicilio es el lugar idóneo para la atención y el cuidado de personas con necesidades paliativas y sus familias^1^, siendo también el lugar en el que la mayor parte de los pacientes desean fallecer^2^. El equipo de atención primaria es el pilar básico de la asistencia domiciliaria y, por lo tanto, el ideal para llevar a cabo no solo la identificación, sino también la atención paliativa^3^. Dispone de accesibilidad, longitudinalidad y conocimiento del paciente (entorno, creencias, necesidades y recursos de apoyo), necesarios para elaborar la planificación de cuidados de forma conjunta.

La estrategia nacional de cuidados paliativos^4^ indica que la mayor parte de las personas con necesidades paliativas deben ser atendidas por los equipos de atención primaria, formando además parte de sus competencias^5^ y siendo necesaria la intervención de los equipos específicos de cuidados paliativos (CP) solo en situaciones de complejidad^6^.

Diversos organismos, como la Oficina Regional Europea de la Organización Mundial de la Salud, ponen de manifiesto la falta de formación en CP en la atención domiciliaria y la necesidad de aumentar la formación de los profesionales^7^. Y en este sentido, la *European Association for Palliative Care* (EAPC)^8^ y la Asociación Española de Enfermería en Cuidados Paliativos (AECPAL)^9^ recomiendan que todos los profesionales de enfermería reciban formación básica en CP en su formación de grado. También, diversos estudios nacionales e internacionales concluyen que existen déficits de formación, y demandan mayor preparación^10^, ^11^, ^12^, ^13^, ^14^, ^15^, ^16^. Además, un estudio con enfermeras hospitalarias ponía de manifiesto que, a pesar de que la mitad de ellas tenían formación básica en CP, solo cerca del 15% los aplicaban en sus cuidados^17^, por lo que dichos conocimientos y cuidados no llegan a los ciudadanos.

Varios instrumentos valoran los conocimientos^18^, ^19^, las competencias^20^ y las actitudes^21^ de las enfermeras hacia el cuidado de pacientes al final de su vida. Sin embargo, solo el cuestionario INCUE^22^ valora específicamente los conocimientos en CP y su aplicación práctica en enfermeras de atención primaria, permitiendo detectar las necesidades de formación en dichas enfermeras.

El objetivo del presente estudio es valorar la formación, la percepción de preparación y las necesidades formativas en CP teóricas y prácticas de las enfermeras de atención primaria en España.

## Material y métodos

### Diseño y población

Se diseñó un estudio descriptivo transversal dirigido a enfermeras de atención primaria del ámbito español.

El tamaño muestral establecido fue de 237 participantes, en base a los datos del Ministerio de Sanidad^23^ (31.159 enfermeras de atención primaria), para una seguridad al 95%, precisión del 3%, proporción p = 0,5 (5%) y pérdidas esperadas del 15%. El muestreo fue no probabilístico, en bola de nieve a través de redes sociales dirigido a enfermeras españolas con asistencia clínica en atención primaria y cuestionario online anónimo, mediante Google Forms, durante enero y febrero de 2021. Se consideraron como criterios de exclusión a las enfermeras pediátricas y las matronas. El reclutamiento mediante líderes naturales, el acceso extendido a internet y el uso profesional de una misma IP permitieron representar la población a estudio, evitaron los sesgos de autoselección y aseguraron la seguridad de los participantes.

### Instrumento

La recogida de datos se realizó a través del instrumento INCUE^22^. Este instrumento evalúa los conocimientos en CP (23 preguntas dicotómicas: sí/no) y su aplicación práctica (30 preguntas) en 5 áreas: principios de los CP, manejo sintomático y planes de cuidados, afrontamiento de pérdida y muerte, habilidades de comunicación y aspectos éticos y legales. La aplicación práctica se mide mediante una escala Likert de 5 puntos (de nunca a siempre). La puntuación mínima para considerar la capacitación teórica se establece en 18 puntos de los 23 aciertos posibles, y la capacitación práctica se establece en 90 puntos de los 120 posibles, puntuando 0 la respuesta «nunca», 1 «raramente», 2 «a veces», 3 «frecuentemente» y 4 «siempre». Otras variables analizadas fueron la formación en CP y la necesidad percibida de preparación y de formación en la materia (mediante respuesta múltiple).

### Análisis estadístico

Se realizaron análisis descriptivos de las características sociodemográficas, personales y profesionales de los/las enfermeros/as. Las variables cuantitativas se resumieron mediante su media y desviación típica. Las variables categóricas se describieron mediante frecuencias y proporciones.

Se analizaron las puntuaciones obtenidas en cada parte del cuestionario, y se compararon los resultados obtenidos según el nivel formativo de los encuestados. También, mediante un test de simetría exacto, si los conocimientos teóricos en cada área de conocimiento se aplicaban en la práctica. Finalmente, se compararon las puntuaciones en la parte teórica y práctica entre los sujetos con un grado y aquellos con un posgrado o formación no universitaria que solo tenían un nivel formativo básico en CP, utilizando el test de Mann-Whitney. Para el análisis de los datos se utilizó el software estadístico R (versión 4.0.2).

### Aspectos éticos

El presente estudio fue aprobado por el Comité de Ética en Investigación de Medicamentos del Hospital Universitario Dr. Peset (Proyecto de Investigación EAPCP19-V01 y código CEIM 11/20). Se proporcionó información y aseguró la confidencialidad a los participantes. Se obtuvo el consentimiento informado por escrito de cada participante, cumpliendo las normas éticas y legales vigentes.

La muestra fue utilizada con anterioridad en la validación del instrumento aplicado.

**Figure:**
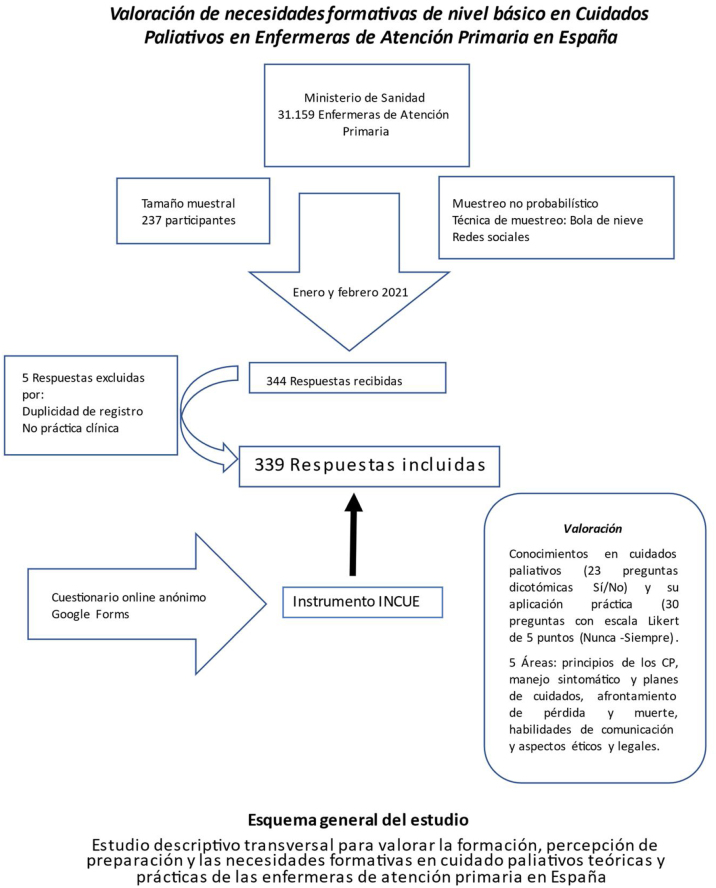
**Esquema general del estudio**.

## Resultados

Se obtuvieron 344 respuestas, de las cuales 339 cumplían criterios de inclusión.

Mayoritariamente eran mujeres, algo más de la mitad eran diplomadas o graduadas, con una media de 21 años de experiencia profesional y desempeñaban tareas como enfermera.

El 86,1% de las enfermeras encuestadas tenían formación en CP, siendo básica en el 45,4% (tabla 1).

**Tabla 1:** Datos sociodemográficos de la muestra

Variables		n = 339	
		Media ± DE	n (%)
Edad (años)		45,5 ± 11,2	
Sexo	Mujer		280 (82,6)
	Hombre		59 (17,4)
Nivel máximo de cualificación profesional	Doctor		17 (5)
	Enfermera especialista		53 (15,6)
	Máster		88 (26)
	Diplomado/Graduado		181 (53,4)
Puesto que desempeña actualmente	Enfermera referente en CP		45 (13,3)
	Enfermera y coordinadora de centro		0 (0)
	Enfermera		208 (61,4)
	Coordinadora de centro		33 (9,7)
	Gestora de casos comunitaria		53 (15,6)
Experiencia profesional en años		21 ± 11,7	
Formación en CP	Sí		292 (86,1)
	No		47 (13,9)
Nivel de formación en CP	Avanzado (máster o doctorado)		51 (15,0)
	Intermedio (80-150 h)		92 (27,2)
	Básico (25-80 h)		154 (45,4)
	NS/NC		42 (12,4)
Como se siente de preparada/o para trabajar con pacientes paliativos	Muy preparado		28 (8,3)
	Bastante		109 (32,2)
	Algo		123 (36,3)
	Poco		75 (22,1)
	Nada		4 (1,2)
Necesidad de mayor formación en CP	Mucho		62 (18,3)
	Bastante		169 (49,9)
	Algo		95 (28)
	Poco		13 (3,8)

CP: cuidados paliativos; DE: desviación estándar; h: horas; NS/NC: no sabe/no contesta.

Solo el 40,5% se sentían bastante o muy preparadas para trabajar con pacientes paliativos, manifestando que necesitaban mucha o bastante formación el 68,2% de las enfermeras.

Las necesidades formativas autopercibidas por los sujetos variaban según su nivel de formación. Así, en las enfermeras con mayor formación predominaban las necesidades de formación en cuidado psicoemocional, espiritual o duelo y afrontamiento de pérdidas, mientras que las que no tenían formación o con formación básica manifestaron necesidades de formación en control sintomático, principios de los CP, psicoemocional o duelo y afrontamiento de pérdidas (tabla 2).

**Tabla 2:** Necesidades formativas autopercibidas según el nivel formativo

Necesidades formativas	Avanzado n = 51 (17,2%)	Intermedio n = 92 (31%)	Básico n = 154 (51,9%)	Sin formación n = 42 (12,4%)
Psicoemocional	56,86%	60,87%	75,97%	45,24%
Duelo y afrontamiento de pérdidas	35,29%	59,78%	68,83%	50%
Control sintomático	31,37%	45,65%	60,39%	88,1%
Habilidades de comunicación	43,14%	41,3%	53,9%	52,38%
Sociofamiliar	27,45%	36,96%	49,35%	54,76%
Aspectos éticos	41,18%	40,22%	48,05%	30,95%
Espiritualidad	56,86%	38,04%	30,52%	33,33%
Principios de los CP	1,96%	10,87%	29,22%	59,52%

CP: cuidados paliativos.

Porcentajes de respuestas del total de respuestas.

Los resultados del cuestionario mostraron que un alto porcentaje superaron la puntuación requerida en la teoría o conocimientos (83,76%), independientemente de su nivel formativo, frente al 43,36% que lo hicieron en la aplicación práctica. Esta misma tendencia en la capacitación se observó en los resultados divididos por nivel de formación y en todas las áreas (tabla 3). Cabe destacar que el área con menor porcentaje de capacitación en todos los niveles de formación fue la de aplicación práctica de afrontamiento de pérdida y muerte.

**Tabla 3:** Proporción de sujetos que superan la puntuación mínima en conocimientos y aplicación práctica, según el nivel de formación

	Avanzado n = 51 (17,2%)		Intermedio n = 92 (31%)		Básico n = 154 (51,9%)		Sin formación n = 42 (12,4%)	
	Bloques							
Áreas	Teórico	Práctico	Teórico	Práctico	Teórico	Práctico	Teórico	Práctico
Principios de los CP	100%	70,59%	96,74%	61,96%	96,75%	40,91%	85,71%	35,71%
Manejo sintomático	96,08%	86,27%	82,61%	65,22%	53,25%	55,84%	30,95%	30,95%
Afrontamiento de pérdida y muerte	92,16%	29,41%	93,48%	33,7%	76,62%	24,03%	64,29%	11,9%
Habilidades de comunicación	94,12%	90,2%	84,78%	78,26%	87,01%	52,6%	69,05%	59,52%
Aspectos éticos y legales	96,08%	80,39%	91,3%	69,57%	94,16%	53,25%	69,05%	38,1%

CP: cuidados paliativos.

**Figura 1:**
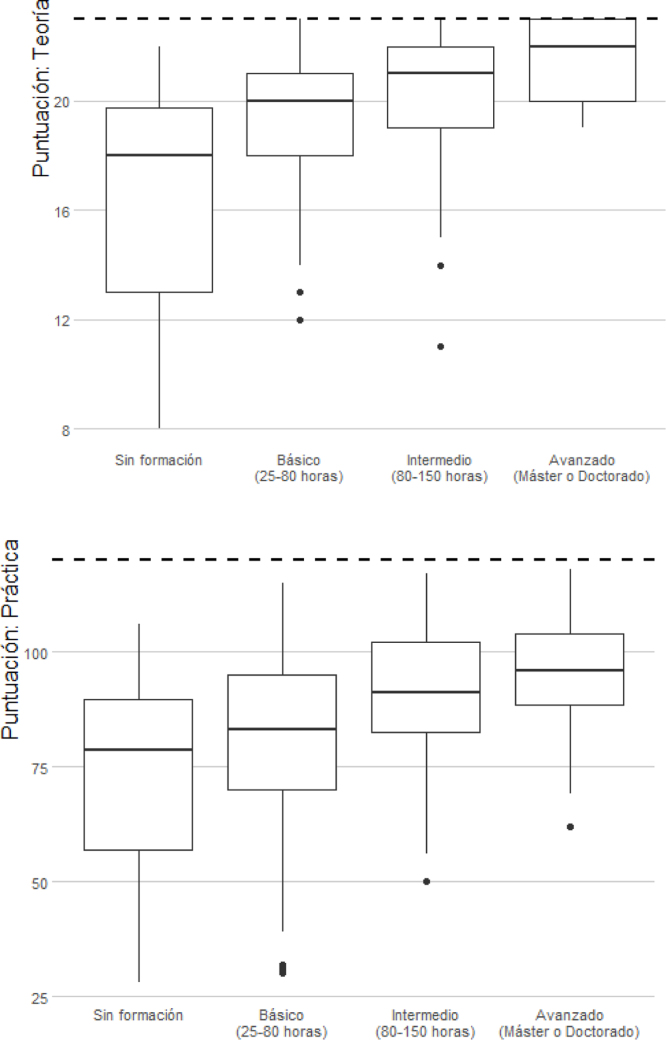
Diagramas de cajas con la distribución de las puntuaciones obtenidas en la parte teórica y práctica del cuestionario según el nivel de formación de los encuestados.

En general, los sujetos con mejores resultados en la parte teórica mostraban también mejores resultados en la parte práctica (fig. 1). La correlación entre las puntuaciones de ambas partes fue positiva y estadísticamente significativa (coeficiente de correlación de Spearman: ρ = 0,43, p ≤0,001).

Se observó que el número de sujetos que superaban la teoría y suspendían la práctica fue significativamente mayor que el número de sujetos que suspendían la teoría y aprobaban la práctica (p ≤ 0,001). Este efecto se observaba también en todas las áreas de conocimiento, excepto en el manejo sintomático y en los planes de cuidados específicos.

**Figura 2:**
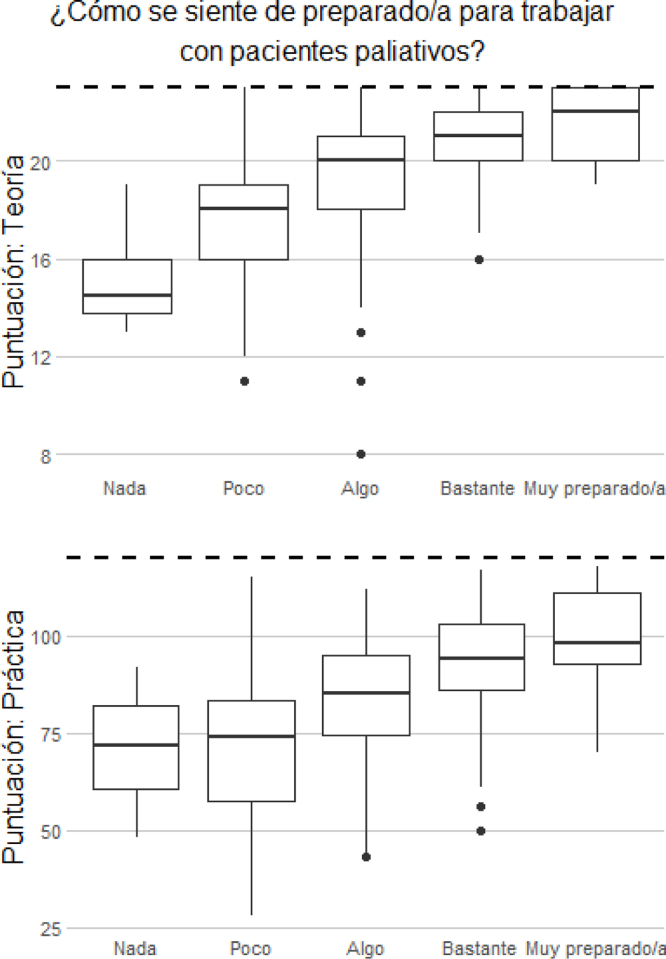
Diagramas de cajas con la distribución de las puntuaciones obtenidas en la parte teórica y práctica del cuestionario según cómo se sienten de preparados los encuestados para trabajar con pacientes paliativos.

Los resultados mostraron que, a mayor formación de los participantes, mayor mediana y mayor puntuación total, con menor dispersión de las puntuaciones y ausencia de valores atípicos. Menor formación implicaba mayor dispersión en las puntuaciones y mayor rango a las respuestas. Esto se observó tanto en la parte teórica como en la parte práctica (Figura 1, Figura 2).

**Figura 3:**
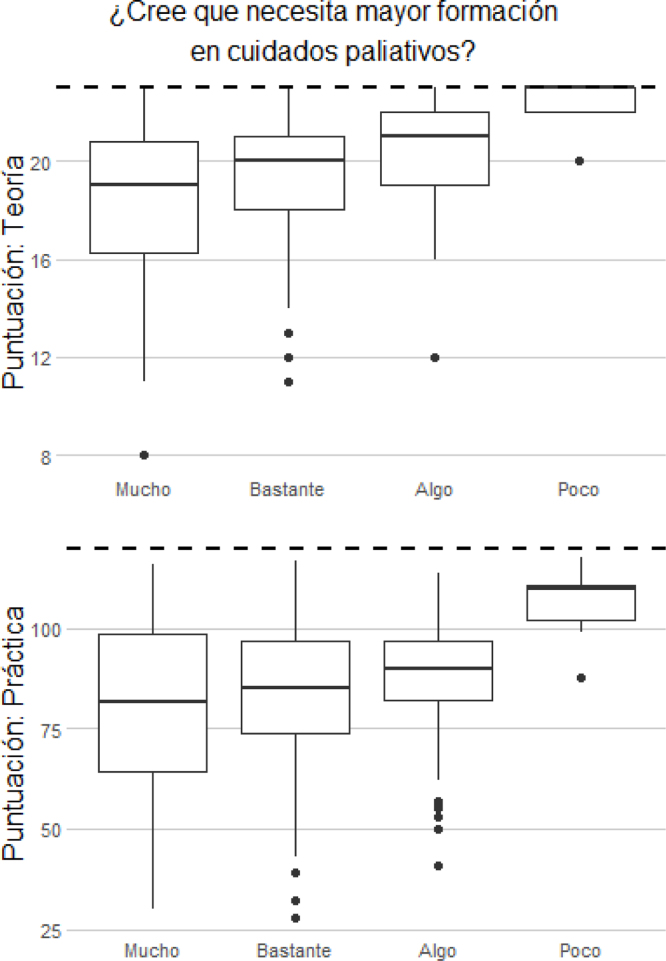
Diagramas de cajas con la distribución de las puntuaciones obtenidas en la parte teórica y práctica del cuestionario según la necesidad de los encuestados de obtener mayor formación en cuidados paliativos.

La mayor percepción sobre la preparación para trabajar en CP se relacionaba positivamente con mejor puntuación tanto en la parte teórica como en la práctica. Esta misma relación también se mantenía en la necesidad de formación, de forma que aquellas que demandaban menor formación eran las que mejor puntuaban en ambos bloques (Figura 2, Figura 3).

Por último, los resultados indicaron que los sujetos con formación básica en CP obtenida en posgrado o en formación no universitaria tenían mayor puntuación que aquellos sujetos con formación en grado, tanto en la teoría (W = 951,5, p ≤ 0,001) como en la práctica (W = 906,5, p ≤ 0,001), y con diferencias estadísticamente significativas entre ambos grupos de sujetos.

## Discusión

La formación en CP de las enfermeras de atención primaria en España es mayor a la que muestran otros estudios en alguna región^10^, otros ámbitos^18^, ^24^, ^25^ y similar a la de otros colectivos^26^. Los resultados obtenidos pueden deberse a la formación avanzada de algunas de las enfermeras, posiblemente porque en algunas comunidades autónomas los equipos específicos de CP están incluidos en los equipos de atención primaria. Por otro lado, la elevada media de edad y, por tanto, la extensa experiencia que tienen las enfermeras, acorde con la media nacional^27^, pueden haber influido también en su formación y en sus conocimientos en CP, tal como también demuestran otros estudios^28^.

Sin embargo, cabe destacar que no todas las enfermeras tienen formación básica como sería necesario y tal como recomiendan diferentes organismos^7^, ^8^, ^9^ e imprescindible para atender en su práctica diaria a las personas con necesidades paliativas y sus familias.

Los porcentajes de enfermeras que no se sienten preparadas para cuidar de personas con necesidades paliativas son superiores a las de otros estudios^24^ y, coherentemente con estos resultados, las enfermeras más preparadas demandan menos formación y obtienen mejores puntuaciones a nivel tanto práctico como teórico. Esto pone de manifiesto que las enfermeras son conscientes de cuáles son sus necesidades formativas.

Al ser un instrumento de reciente creación, no ha sido posible comparar los resultados de la muestra con los de otros estudios, sobre todo a nivel práctico. Los conocimientos en CP demostrados por los sujetos son mayores a los de otros estudios (83,76% frente a 66,7-41,65%), medidos con otros instrumentos (PCQN y Rotterdam MOVE2PC) y que contemplan solo algunas de las áreas, pero no todas^18^, ^19^. Esta variación en el instrumento de medida podría haber influido en los resultados.

Las preguntas del cuestionario son de nivel básico, por lo que todas las enfermeras deberían haber puntuado de forma similar. Sin embargo, las enfermeras con formación intermedia o avanzada puntuaron mejor que las de formación básica. Posiblemente las de mayor formación hayan necesitado formarse a raíz de los retos presentados en su práctica clínica. Esto hace cuestionarse cuál debería ser la carga lectiva para obtener una formación básica. Debería ser suficiente para adquirir las competencias teóricas, pero también para su traslado a la práctica.

Por otro lado, parece que la formación de posgrado en CP es más efectiva que la incluida en el grado. Estos resultados pueden justificarse con la falta de uniformidad y de obligatoriedad de la formación dentro del grado de enfermería en nuestro país^15^, por lo que la búsqueda de formación de posgrado venga impulsada por la necesidad o por el interés en el tema.

Por bloques, muchos más sujetos aprueban la teoría que la práctica, al igual que en el estudio realizado en un hospital de agudos^21^, confirmando que no siempre los conocimientos adquiridos en la teoría tienen traducción en la práctica, y sin repercusión en el cuidado del paciente y su familia. Otros estudios también ponen de manifiesto la escasa repercusión en el cuidado de estos pacientes en atención primaria, con solo alrededor del 50% de planes de cuidados hechos o de visitas domiciliarias de enfermería^9^.

Cabe resaltar la discrepancia de resultados en el área de afrontamiento de pérdida y muerte, entre capacitados en teoría y práctica, incluso en los que tienen un nivel avanzado de formación en CP. Todo ello cuando una de las competencias profesionales de atención primaria es la atención al duelo y la prevención del duelo disfuncional^5^.

Estos resultados muestran la falta de formación básica en CP de las enfermeras de atención primaria, ámbito en el que muchos pacientes con necesidades paliativas y sus familias deben ser cuidados. Además, deberían iniciar una reflexión sobre la falta de traslado de los conocimientos a la aplicación práctica de ellos.

## Limitaciones

El diseño de la investigación contempló los posibles sesgos de autoselección de los participantes derivados del tipo de muestreo y la difusión del cuestionario a través de redes sociales, al utilizar estrategias de reclutamiento a través de líderes naturales (sociedades científicas de enfermería en atención primaria) para obtener una adecuada representación de toda la población a estudio. No fue considerado el rastreo de IP de los participantes, dado que ello no garantiza la respuesta de un mismo sujeto en dos direcciones IP. Además, en un contexto profesional una misma IP puede ser utilizada por diferentes profesionales.

## Conclusiones

La formación básica enfermera en CP y su aplicación práctica en atención primaria continúa siendo una asignatura pendiente; su déficit es percibido, y constituye una de las demandas de las enfermeras, sobre todo en áreas de control sintomático, psicoemocional o atención al duelo. Es necesaria una formación dirigida que repercuta directamente en la aplicación de los cuidados de las personas con necesidades paliativas y sus familias a nivel de la comunidad, y no quede solo en conocimientos.

Posteriores estudios podrían fortalecer los resultados obtenidos y detectar necesidades formativas en áreas sanitarias o grupos específicos que permitieran diseñar y realizar formación específica, dirigida según las necesidades detectadas.

## Consideraciones éticas

El estudio se realizó de acuerdo con las directrices de la Declaración de Helsinki y fue aprobado por el Comité de Ética en Investigación de Medicamentos del Hospital Universitario Dr. Peset (código CEIM 11/20)

## Financiación

El presente trabajo ha obtenido una ayuda en la I Convocatoria de Ayudas de I+D+i en Enfermería 2019 (UGP-19-258) de la Fundació per al Foment de la Investigació Sanitària i Biomèdica de la Comunitat Valenciana (Fisabio). Los promotores colaboraron en el análisis estadístico y en la interpretación de los datos como asesores metodológicos.

## Conflicto de intereses

Los autores declaran no tener ningún conflicto de intereses.
